# Seroprevalence of *Getah virus* in pigs in Southeast China determined with a recombinant Cap protein-based indirect ELISA

**DOI:** 10.3389/fmicb.2025.1547670

**Published:** 2025-02-17

**Authors:** Jianhui Lan, Leilei Duan, Xuya Liu, Yao Zhou, Botao Zeng, Siya Chen, Yu Ye, Dongyan Huang, Gen Wan, Fanfan Zhang, Deping Song

**Affiliations:** ^1^College of Animal Science and Technology, Jiangxi Agricultural University, Nanchang, China; ^2^Jiangxi Engineering Research Center for Animal Health Products, Nanchang, China; ^3^Institute of Animal Husbandry and Veterinary Medicine, Jiangxi Academy of Agricultural Sciences, Nanchang, China

**Keywords:** *Getah virus*, seroprevalence, IgG, ELISA, swine

## Abstract

*Getah virus* (GETV) is a mosquito-borne virus with a broad host range, including mosquitoes and various animals such as pigs, horses, goats, cattle, boars, and blue foxes. In pigs, GETV can cause fever, abortion and reproductive disorders in sows, as well as fever, tremors, and diarrhea in piglets, posing a serious threat to the pig production industry. However, there are few reports on the epidemiology of GETV in China, and reliable diagnostic kits for large-scale detection of GETV antibodies are lacking. Therefore, this study aimed to establish a rapid, sensitive and suitable GETV antibody detection assay for clinical detection. We expressed the recombinant protein Cap in *Escherichia coli* by constructing a recombinant plasmid, pET-32a-Cap, which contained a His tag and the GETV-Cap domain. The expression of the recombinant protein was achieved in the supernatant following bacterial lysis by optimizing the culture temperature and duration for *Escherichia coli*. The recombinant Cap protein was successfully purified via a nickel affinity column, which was used for develop an indirect ELISA method (rCap-ELISA). Following optimization of the rCap-ELISA reaction conditions, a cutoff value of 0.45 was established with 100 swine serum samples analyzed by indirect immunofluorescence (IFA). The overall coincidence rate between rCap-ELISA and IFA was 95.83%, with a 94.03% sensitivity and 100.00% specificity. IgG antibodies against GETV were subsequently detected in 2,102 serum samples from pig farms in Jiangxi and Fujian provinces via the rCap-ELISA method, and the positive rates were 63.36% (1,102/1739) and 37.1% (137/363), respectively. The findings suggest that the indirect ELISA method (rCap-ELISA) is a reliable, accurate, and cost-effective way to detect IgG antibodies against GETV in pigs. This technique is valuable for understanding the dynamics of GETV transmission and for preventing GETV epidemics in pigs.

## Introduction

*Getah virus* (GETV) is taxonomically classified within the genus *Alphavirus* and the family *Togaviridae* (ICTV, 2019). It is the first alphavirus to be isolated and is a typical arbovirus in China. Horses and pigs are the primary animals affected by GETV, but it can infect a variety of species, including blue foxes, cattle, and goats (Kuwata et al., 2018; Liu et al., 2019; Bannai et al., 2015; Zhai et al., 2008). In horses, GETV can cause fever, rash, and hindlimb edema (Kamada et al., 1991). In pigs, it can cause fever, anorexia, depression, abortion and reproductive disorders in sows (Izumida et al., 1988), and clinical symptoms such as reduced appetite, fever, and tremors in piglets (Yago et al., 1987). In humans, antibodies against GETV had been detected in serum (Fukunaga et al., 1981; Lu et al., 2020). Due to its adaptability, GETV has spread from its original isolation in Malaysia in 1955 (MM2021) to cold countries like Russia and Mongolia, subtropical areas like China and Japan, and tropical regions like the Philippines, India, and Pacific island states (Morita and Igarashi, 1984; Li et al., 2022). In Hainan Province, China, GETV (M1) was initially discovered from *Culex* mosquitoes in 1964 (Li et al., 1992). By 2018, GETV had affected 16 provinces in China, and this number had risen to 21 by 2022, continuing to increase. In 2017, a GETV (HuN1) outbreak on a pig farm in Hunan resulted in piglets dying 5–10 days after birth and sows having stillbirths or mummified fetuses (Yang et al., 2018). The increasing incidence of GETV in China poses a serious threat to global public health and the swine industry.

The genome of GETV is a single-stranded, positive-sense RNA ranging from 11,000 to 12,000 nucleotides (nt) in length. The viral particles are spherical, approximately 70 nm in diameter, with an envelope and protrusions, and can agglutinate red blood cells from animals such as horses, pigs, sheep, and humans (O type) (Kamada et al., 1982). According to the phylogenetic analyses, the complete genome of GETV strains are divided into four groups (I-IV), with most Chinese isolates belonging to Group III. The GETV genome has a methylated cap structure at the 5′ terminus and a poly(A) tail structure at the 3′ terminus, encoding two large open reading frames (ORFs). The ORF at the 5′ terminus encodes four nonstructural proteins (NSP1, NSP2, NSP3 and NSP4) (Pfeffer et al., 1998), and the ORF at the 3′ terminus encodes five structural proteins, including the Cap protein, E3 protein, E2 protein, 6 K protein and E1 protein (Kobayashi et al., 2016). The Cap gene is 804 nt and encodes 268 amino acids, playing an indispensable role in the assembly and replication of the virus (Strauss and Strauss, 1994; Ferreira et al., 2003; Sun et al., 2024). The Cap protein is highly conserved and immunogenic, making it a promising candidate for detecting GETV antibodies.

Currently, various molecular biological techniques are available for the detection of GETV, including RT-PCR, RT-qPCR, loop-mediated isothermal amplification (LAMP), indirect immunofluorescense (IFA), and virus neutralization test (VNT). However, these methods are not suitable for large-scale clinical testing. For example, the RT-PCR method is simple and cost-effective but has poor specificity and is prone to nonspecific amplification, leading to false positives, and it cannot perform quantitative analysis of samples. While the qPCR and LAMP methods offer high sensitivity, they are more expensive and require more sophisticated experimental environments and equipment, limiting their broader application. Conventional methods for isolating and identifying the virus can detect GETV accurately; however, they require a professional laboratory, which is difficult to obtain (Xing et al., 2020). An efficient and inexpensive way is to conduct serological surveys via ELISA to investigate the epidemiological dynamics of GETV. The advantages of this method are its speed, sensitivity, and affordability, which make it suitable for large-scale epidemiological surveillance. Additionally, after pigs recover from GETV infection, although the viral particles and antigenic materials in the body have been cleared by the immune system, antibodies persist for a long period, allowing for a longer detection window and providing more information on past infections. In 2012, Japanese researchers developed an ELISA using antigens extracted from GETV-infected Vero cells and detected GETV antibodies in wild boars in Japan (Kuwata et al., 2018). In 2020, synthetic peptides of the E2 protein were used as antigens to detect the serum of horses artificially infected with GETV, resulting in good results (Bannai et al., 2019). These studies indicate that ELISA is a viable method for detecting GETV antibodies.

In this study, we developed a new indirect ELISA by using the recombinant prokaryotic expressed GETV-rCap protein. This method allows for the rapid, accurate, and efficient detection of IgG antibodies against GETV in pigs, providing a valuable diagnostic tool for the serological surveillance of GETV infection. Furthermore, we evaluated the prevalence of GETV in southern China by analyzing 2,102 pig serum samples collected from 2018 to 2024 using the rCap-ELISA method.

## Materials and methods

### Virus, cell culture, and serum sample collection

The porcine Getah virus strain GETV-JX-CHN-22-P7 (GenBank No. 863732) was isolated from a GETV-affected piglet in Jiujiang, Jiangxi province, China. The Vero cell line CCL-81 was obtained from the American Type Culture Collection (ATCC, United States) and was maintained in DMEM supplemented with 10% fetal bovine serum (FBS; Gibco, Australia) and 100 U/mL penicillin and 100 μg/mL streptomycin at final concentrations. Sera positive for porcine epidemic diarrhea virus (PEDV), porcine reproductive and respiratory syndrome virus (PRRSV), pseudorabies virus (PRV), and classical swine fever virus (CSFV) were obtained from our laboratory. Serum samples were systematically collected from commercial pig farms across various regions in Jiangxi and Fujian Provinces in South China in September 2018 and from October 2023 to May 2024.

### Construction of the recombinant expression plasmid

**Figure 1:**
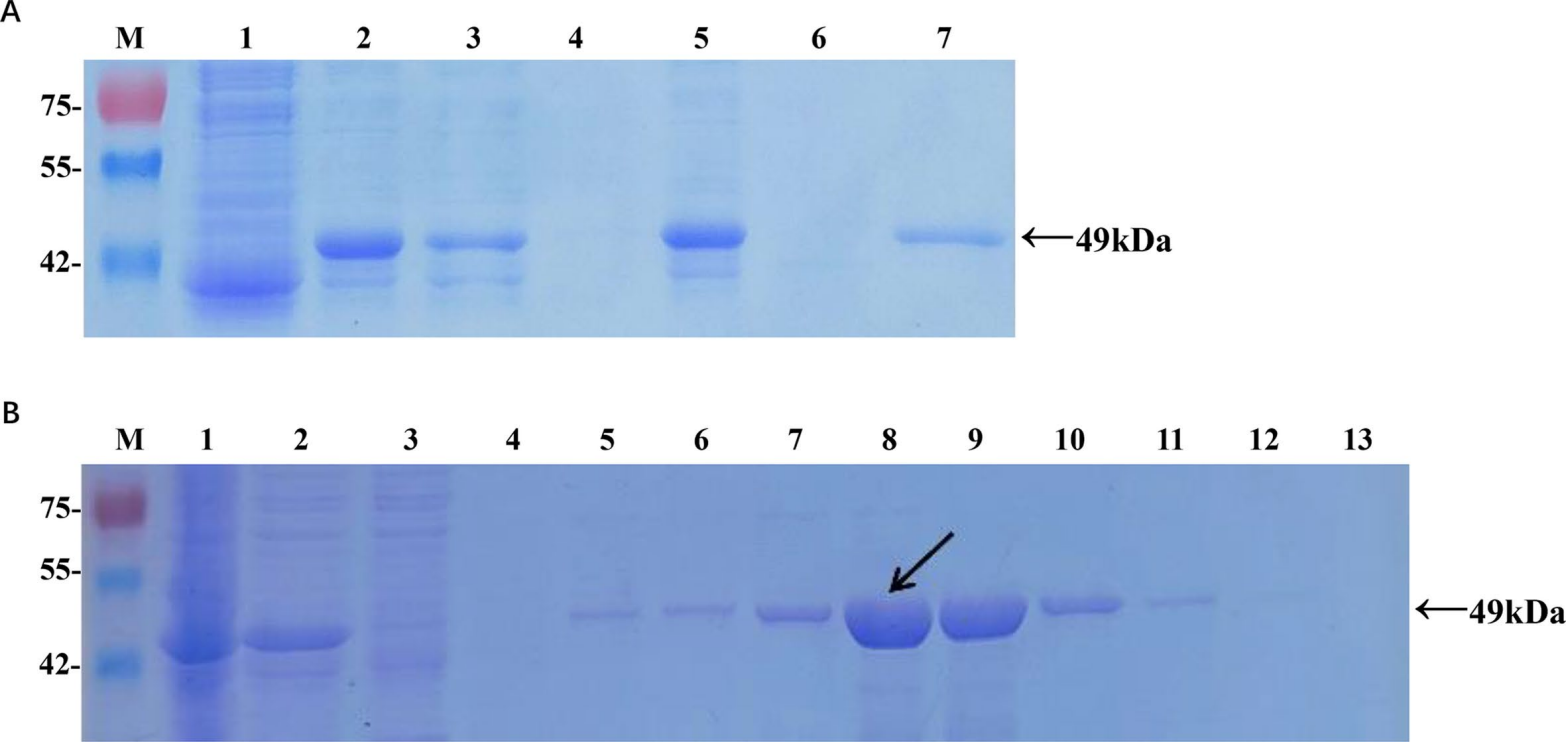
Expression and purification of the recombinant Cap protein in *Escherichia coli*. **(A)** Analysis of soluble recombinant Cap protein. M, protein marker; lane 1, cell lysate from pET-32a at 16°C; lane 2, cell lysate from pET-32a-Cap at 16°C; lane 3, supernatant from pET-32a-Cap at 16°C; lane 4, pellet from pET-32a-Cap at 16°C; lane 5, cell lysate from pET-32a-Cap at 37°C; lane 6, supernatant from pET-32a-Cap at 37°C; lane 7, pellet from pET-32a-Cap at 37°C. **(B)** Optimization of the imidazole concentration for the elution. M, protein marker; lane 1, cell lysate from pET-32a-Cap; lane 2, supernatant from pET-32a-Cap; lane 3, flow-through; lane 4, wash; lanes 5–13, 20 mM, 50 mM, 100 mM, 150 mM, 200 mM, 250 mM, 300 mM, 400 mM, and 500 mM imidazole in the elution buffer. The black arrow indicates the optimal concentration.

To facilitate the genetic characterization of the Cap gene, a conserved element analysis was conducted on 78 reference sequences retrieved from the National Center for Biotechnology Information (NCBI) using MEGA 11.0 software. Genomic RNA from the GETV-JX-CHN-22-P7 strain (GenBank accession number OQ863732) served as the template for this analysis. The coding region of the Cap gene was amplified from the genomic RNA of strain GETV-JX-CHN-22-P7. The primers used were as follows: forward primer, 5’-CCG*gaattc*ATGAATTACATTCCAACTCA-3′ (the italic lowercase letters indicate the cleavage site of restriction enzyme *EcoRI*); reverse primer, 5’-CCG*ctcgag*TTACCATTCTTCTGTCCCTTC-3′ (the italic lowercase letters indicate the cleavage site of restriction enzyme *XhoI*). The underlined and italicized portions represent the *EcoRI* and *XhoI* restriction sites, respectively. The amplification product, confirmed by agarose gel electrophoresis to be 804 bp, was cloned and inserted into the *EcoRI* and *XhoI* restriction sites of the prokaryotic expression vector pET-32a. The recombinant plasmid was subsequently designated pET-32a-GETV-Cap (Figure 1).

### Expression and purification of the recombinant cap protein

The conditions for inducing the expression of the recombinant Cap protein were optimized as follows: 0.6 mM isopropyl *β*-D-1-thiogalactopyranoside (IPTG) was used in LB medium, and the culture was incubated at 16°C for 16 h. The recombinant protein was then extracted and purified using a His-tag affinity purification kit (Sangon, Shanghai, China). Elution from the nickel affinity column was performed with 150 mM imidazole, a concentration determined to be optimal through empirical testing. To facilitate the correct folding of the recombinant protein, a dialysis process was conducted in 0.01 M phosphate-buffered saline (PBS) at 4°C for 3 h. The purified recombinant Cap protein was validated by SDS-PAGE, and its concentration was subsequently quantified using a BCA protein assay kit (Yasen, Shanghai, China).

To produce antibodies target Cap protein, five 7-week-old female BALB/c mice were used in the immuni-zation experiment. First, 200 uL of blood was collected from each mouse to separate the serum, which was used as the negative control for subsequent antibody detection. Recombinant Cap protein and the same amount of complete Freund’s adjuvant (SEPPIC, Shanghai, China) were used as antigens after being completely emulsified and mice were immunized by subcutaneous multi-point injection. On the 14th, 28th, and 42th days, the mice were immunized with equal amounts of rCap and Freund’s incomplete adjuvant. The inoculated mice were kept in good conditions during the experiment, and blood samples were collected on the 49th day. The blood was centrifuged at 4000 rpm for 5 min at 4°C to separate the antiserum. The titer of purified antibodies was measured using an enzyme-linked immunosorbent assay (ELISA).

### Development of the rCap-based indirect ELISA (rCap-ELISA)

A common indirect ELISA for detecting serum antibodies was established as previously described. Briefly, 100 μL of rCap, gradually diluted from 12.5 to 1.25 μg/mL in phosphate-buffered saline (pH 9.6), was coated on 96-well ELISA plates (Jet Bio-Filtration, China) and incubated overnight at 4°C. After washing, the plates were blocked with 300 μL/well of different blocking buffers, including bovine serum albumin (BSA) or skim milk (SM), and incubated for 60 to 150 min at 37°C. Subsequently, 100 μL/well of pig serum, diluted from 1:50 to 1:6400, was added, and the mixture was incubated for 30 to 60 min at 37°C. Next, the plates were incubated with 100 μL/well of secondary antibody (Rabbit Anti-Pig IgG H&L; Solarbio, China) at dilutions ranging from 1:500 to 1:4000 for 30 to 90 min at 37°C. To visualize the peroxidase reaction, 100 μL of 3,3′,5,5′-tetramethylbenzidine (TMB; Solarbio, China), as the substrate for HRP, was added to each well and incubated for 10 to 20 min at 25°C in complete darkness, followed by the addition of 50 μL of 2 M H2SO4 (Solarbio, China). Each well was washed three times with 200 μL of 1 × PBST (1 × PBS containing 0.05% Tween-20) before the addition of blocking buffer, serum, antibody, or substrate. Finally, the OD_450_ value of each well was measured and recorded immediately using a Thermo Scientific™ Varioskan™ LUX microplate reader (Applied Biosystems, United States). The reaction conditions were deemed optimal when the OD_450_ ratio between the positive and negative sera (P/N value) reached its peak, and the OD_450_ value of the positive serum approached 1.0, while that of the negative serum was minimized.

### Determination of the cutoff value of the rCap-ELISA

Based on results from 100 clinical serum samples (50 seronegative and 50 seropositive confirmed by IFA) using the rCap-ELISA method, receiver operating characteristic (ROC) curves were generated, and the area under the curve (AUC) was calculated. The sensitivity (Se) and specificity (Sp) of the ELISA were also determined. The Youden index (Youden = Se + Sp - 1) was employed to identify the optimal cutoff value for ELISA, where a sample with an OD_450_ equal to or greater than the cutoff value was considered positive.

### Sensitivity, specificity, and repeatability of the rCap-ELISA

To assess the sensitivity of the rCap-ELISA, twofold serum dilutions ranging from 1:400 to 1:3,200 were tested. Four positive sera against FMDV, PEDV, PRRSV, PRV, tetanus, and Salmonella were used to verify the specificity of the method. Intra-assay repeatability was evaluated by testing GETV-positive serum in six replicates on the same ELISA plate.

### Detection of clinical pig sera via rCap-ELISA and IFA

To further investigate the prevalence of GETV in pigs in Jiangxi, the ELISA method developed in this study was used to test 96 clinical serum samples from pig farms. The results were compared with those obtained via the IFA method to determine the coincidence rate. For IFA observation, Vero-81 cells were seeded in 96-well plates and infected with GETV-JX-CHN-22-P7 at a multiplicity of infection (MOI) of 0.01. At 24 h post-infection, the cells were washed three times with PBS and fixed with 4% paraformaldehyde for 30 min at 4°C. After another three washes with PBS, the cells were permeabilized with cold methanol for 15 min at −20°C and subsequently blocked with 5% bovine serum albumin (BSA) at 37°C for 1 h. The cells were then incubated with pig serum samples (dilution 1:100) for 1 h at 37°C. Following three more washes with PBS, the cells were incubated with FITC-conjugated goat anti-pig IgG (H + L) (dilution 1:500) (Solarbio, China) for 30 min at 37°C. The cells were washed three times with PBS and counterstained with DAPI (dilution 1:1,000) (Boster, Wuhan, China) at room temperature for 15 min. After a final wash with PBS, all wells were examined using fluorescence microscopy (Ax10 Axio, Zeiss, Germany). Specific fluorescence in the cytoplasm and cellular space was considered positive.

### Statistical analysis

To analyze the relationship between rCap-ELISA and IFA, we calculated the coefficient of variation (CV), receiver operating characteristic (ROC) curves, and correlation coefficients using Excel and GraphPad Prism 8.3.0 software. The concordance rate was calculated as follows: Coincidence rate = (number of true positive samples + number of true negative samples) / total number of samples. The chi-square test was used to compare the differences in positive rates across different areas. The kappa value was assessed using SPSS26.0.

## Results

### Expression and purification of the GETV-cap protein

The recombinant plasmid pET-32a-Cap was constructed according to the manufacturer’s instructions (Supplementary Figure S1). Protein expression in positive colonies was induced by adding 0.6 mM IPTG, followed by incubation at 16°C for 16 h (Figure 1A). After purification and dialysis, samples of the recombinant Cap protein (rCap) were analyzed using SDS-PAGE (Figure 1B). We confirmed that the recombinant protein, approximately 49 kDa, was expressed mainly as a soluble protein and could be successfully purified using a nickel affinity column with 150 mM imidazole. The concentration of the renatured protein was approximately 0.87 mg/mL.

### Establishment of cap-ELISA for detecting GETV antibodies

**Figure 2:**
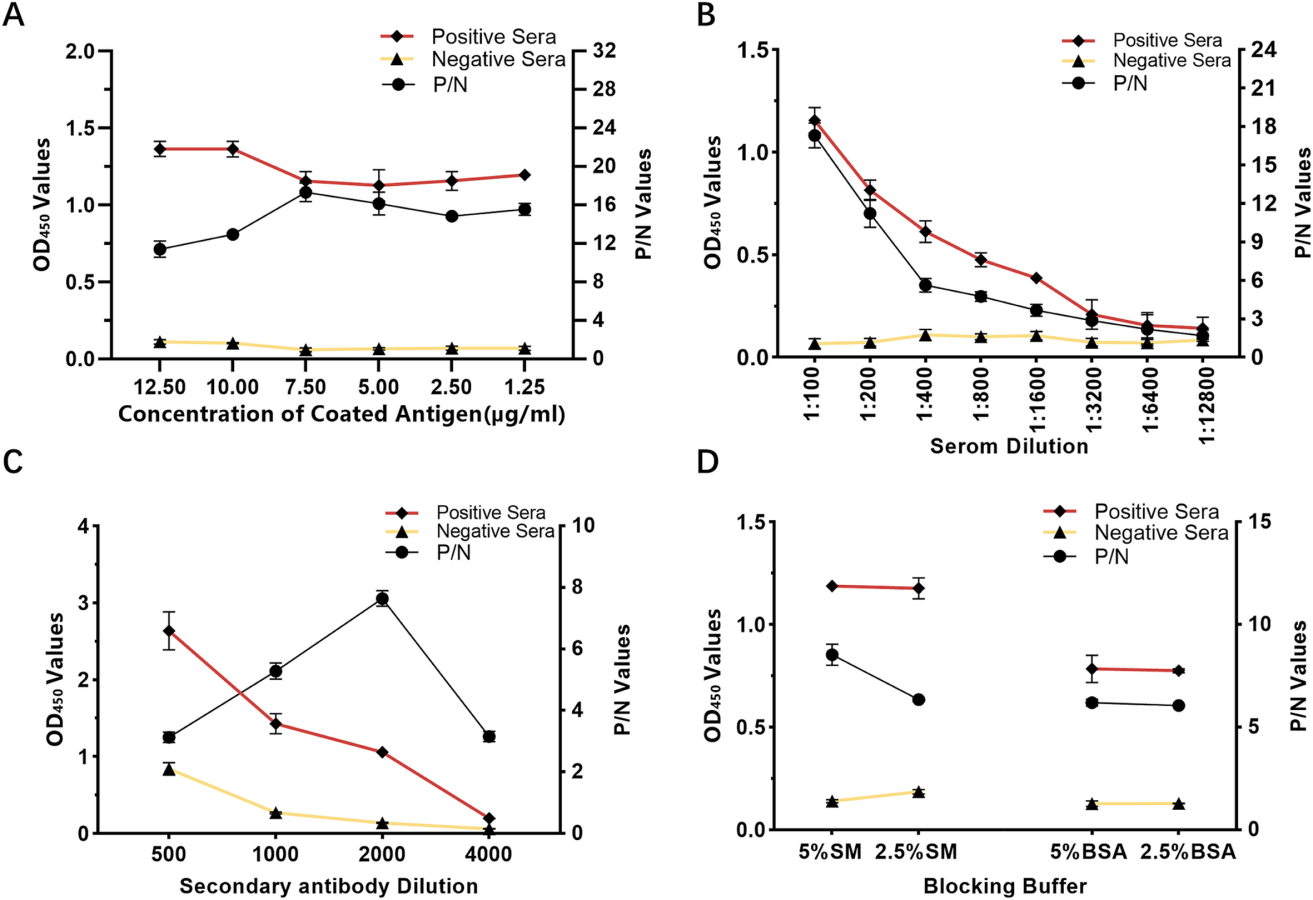
Optimization of rCap-ELISA working conditions. **(A)** Optimization of the coating antigen concentration. **(B)** Determination of the appropriate serum sample dilution. **(C)** Selection of the optimal blocking buffer. **(D)** Determination of the optimal dilution for the secondary antibody (HRP-labeled rabbit anti-pig IgG). Conditions were considered optimal when the OD_450_ ratio between positive and negative sera (P/N value) was highest, with an OD_450_ value of the positive serum close to 1.0 and the negative serum as low as possible.

The conditions for antigen protein coating were optimized at different concentrations, temperatures and times. The optimal working concentration of antigen was 7.5 μg/mL and the appropriate serum dilution was 1:100 (Table 1; Figure 2A). The optimal dilutions for the serum and secondary antibodies were found to be 1:100 and 1:2000, respectively (Figures 2B,C). Additionally, 5% skim milk (SM) was identified as the best blocking buffer after 2 h of incubation at 37°C (Figure 2D). The optimal reaction conditions for the rCap-ELISA were 60 min for both the serum and secondary antibody, and 15 min for the substrate (Supplementary Figures S2A–D).

**Table 1:** Determination of the optimal protein coating concentration and serum dilution.

Serum dilution		Protein coating Concentration (μg/mL)					
		12.5	10.0	7.5*	5.0	2.5	1.25
1:50	P	1.995	1.701	1.551	1.021	0.992	0.759
	N	0.189	0.155	0.121	0.099	0.089	0.071
	P/N	10.556	10.974	12.818	10.313	11.146	10.690
1:100*	P	1.365	1.364	1.156	1.128	1.157	1.196
	N	0.12	0.105	0.067	0.07	0.078	0.077
	P/N	11.409	12.935	17.319*	16.158	14.845	15.563
1:200	P	0.875	0.805	0.815	0.681	0.699	0.647
	N	0.082	0.091	0.073	0.054	0.061	0.111
	P/N	10.699	8.853	11.23	12.587	11.434	5.852
1:400	P	0.744	0.579	0.613	0.651	0.469	0.504
	N	0.097	0.091	0.109	0.07	0.066	0.069
	P/N	7.637	6.372	5.621	9.321	7.053	7.253
1:800	P	0.542	0.528	0.476	0.486	0.424	0.441
	N	0.122	0.111	0.1	0.096	0.092	0.077
	P/N	4.434	4.775	4.742	5.067	4.626	5.756
1:1600	P	0.364	0.359	0.387	0.305	0.282	0.264
	N	0.13	0.117	0.105	0.081	0.096	0.072
	P/N	2.803	3.063	3.676	3.753	2.933	3.684
1:3200	P	0.209	0.214	0.209	0.151	0.163	0.139
	N	0.091	0.093	0.073	0.069	0.058	0.065
	P/N	2.287	2.299	2.87	2.198	2.81	2.128
1:6400	P	0.179	0.174	0.156	0.149	0.125	0.16
	N	0.089	0.085	0.071	0.069	0.062	0.068
	P/N	2.011	2.037	2.185	2.151	2.012	2.36
1:12800	P	0.154	0.235	0.142	0.112	0.116	0.078
	N	0.095	0.085	0.084	0.07	0.076	0.091
	P/N	1.618	2.775	1.693	1.6	1.54	0.855

P, positive serum; N, negative serum. Optimal conditions selected the maximum P/N value. The OD values presented are average values. “*” means the optimum conditions.

### Cutoff value and specificity of the rCap-ELISA

**Figure 3:**
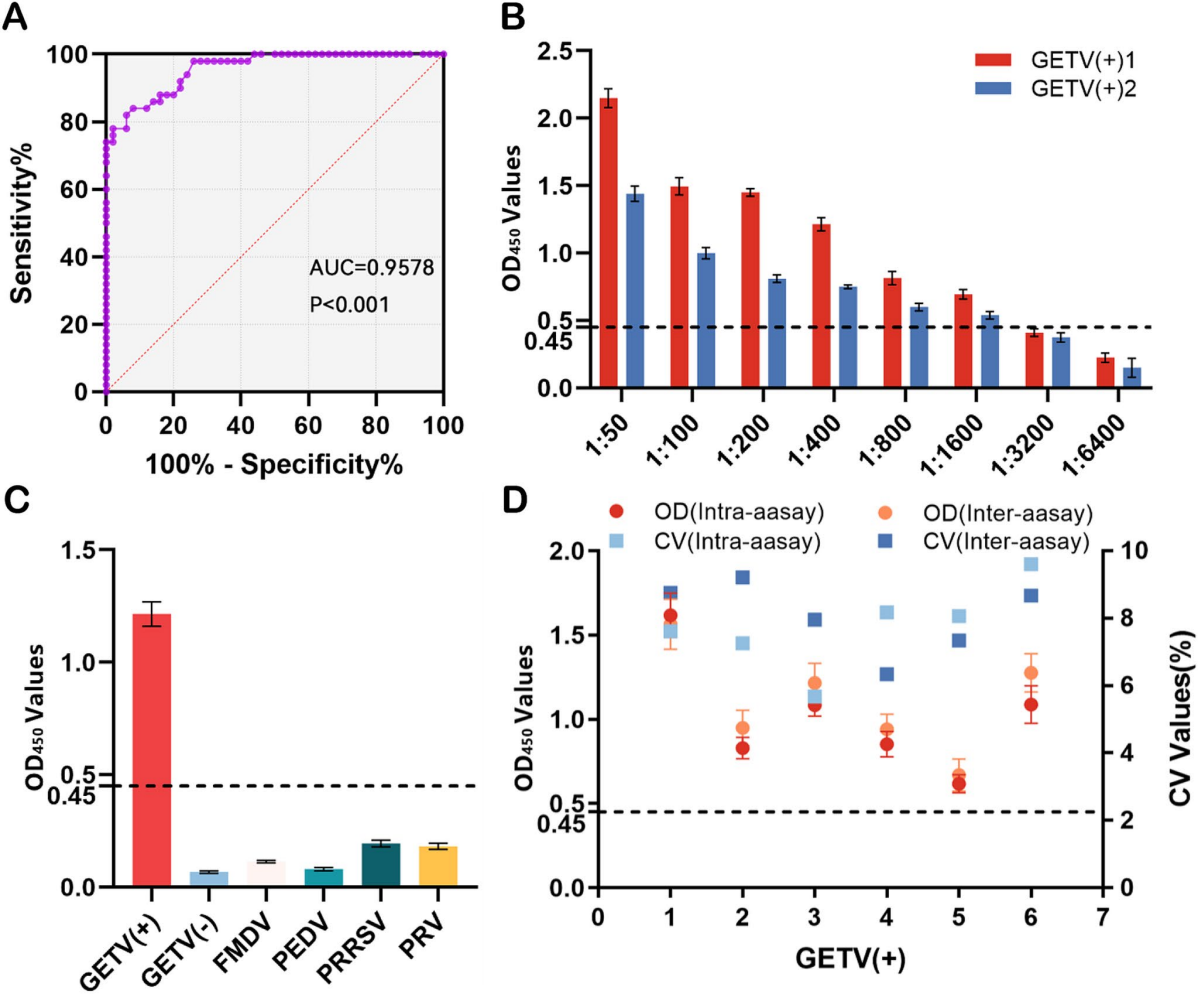
Validation of the indirect ELISA. **(A)** Receiver operating characteristic (ROC) curve for the rCap-ELISA, based on results from rCap-ELISA and IFA with 100 serum samples. The purple dots represent the test curve and the red dashed line corresponds to the noninformative test curve. The area under the ROC curve (AUC) of the developed ELISA was 0.958. **(B)** Sensitivity testing using eight dilutions of GETV-positive serum. **(C)** Specificity testing using swine-positive sera against foot-and-mouth disease virus (FMDV), porcine epidemic diarrhea virus (PEDV), porcine reproductive and respiratory syndrome virus (PRRSV), and pseudorabies virus (PRV) via rCap-ELISA, calculating the mean OD450 value to determine positivity. **(D)** Repeatability testing using six GETV-seropositive samples via rCap-ELISA, calculating the OD450 values and CV values for intra- and interassay reproducibility. The dashed line indicates the cutoff value (0.45) for the rCap-ELISA.

To determine the cutoff value of the rCap-ELISA, 50 seronegative and 50 seropositive samples (verified by IFA) were tested. ROC curve analysis showed an AUC value of 0.958, with the highest Youden index of 0.76, calculated from a sensitivity of 0.84 and specificity of 0.92. Based on the highest Youden index, the cutoff value was set at 0.45, indicating that a sample with an OD450 value ≥0.45 is considered GETV seropositive (Figure 3A).

### Specificity, sensitivity, and repeatability of the rCap-ELISA

Sensitivity analysis indicated that the method had good sensitivity, as the standard GETV-positive serum reacted clearly up to a titer of 1:1600 (Figure 3B). To assess potential cross-reactivities of the rCap protein with antibodies against common swine pathogens, sera positive for FMDV, PEDV, PRRSV, and PRV, as well as tetanus and Salmonella, were tested using rCap-ELISA. The results showed weak reactions with sera positive for FMDV, PEDV, PRRSV, and PRV, with values below the cutoff of 0.45, specifically 0.114, 0.081, 0.195, and 0.182, respectively (Figure 3C). In repeatability experiments, the intra-assay and inter-assay coefficients of variation (CVs) for the rCap-ELISA were determined using five GETV-positive serum samples, resulting in values of 5.68–9.60% and 6.34–9.21%, respectively (Figure 3D).

### Detection of clinical pig sera via rCap-ELISA and IFA

**Figure 4:**
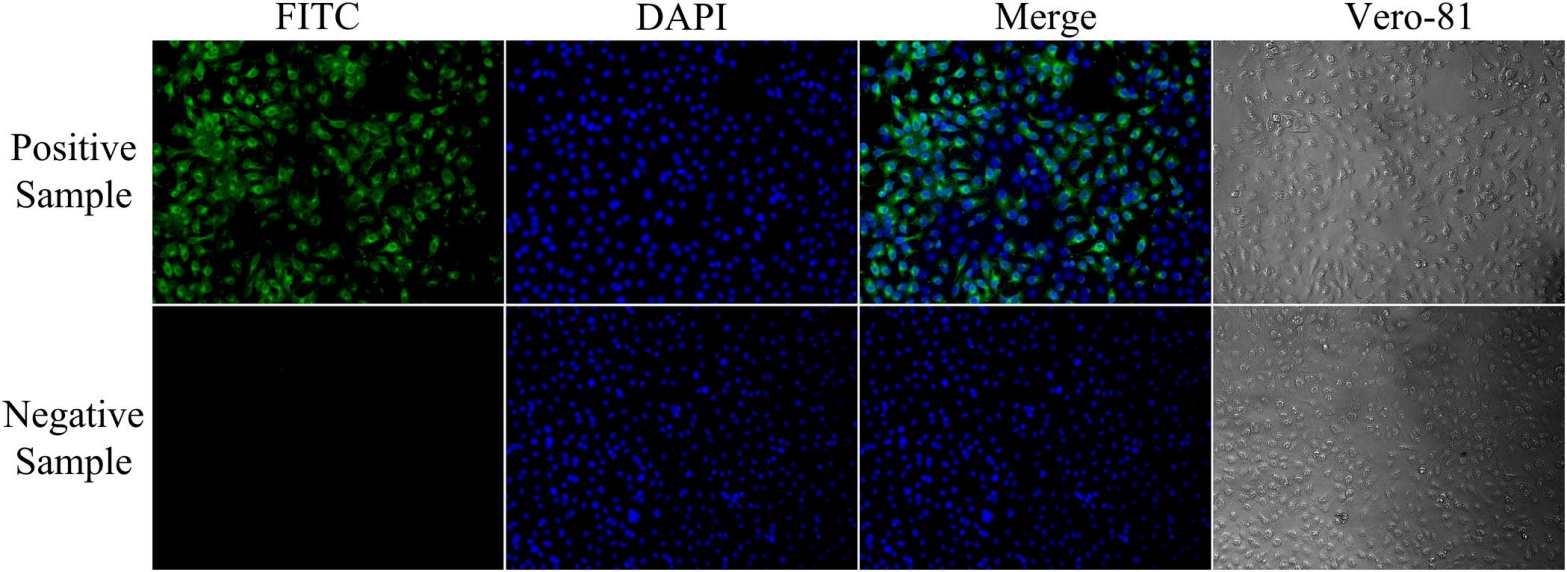
Identification of GETV-negative and positive sera by indirect immunofluorescence assay. Negative serum samples showed no visible fluorescence in the immunofluorescence assay. Strongly positive serum samples showed high-intensity fluorescence (200× magnification).

The results of the rCap-ELISA method revealed a total positive rate of 65.62%, whereas the rate for neutralizing antibodies determined by IFA was 69.79% (Table 2; Figure 4). When 96 samples were tested using both ELISA and IFA, ELISA identified 4 false positive samples. Additionally, the validated rCap-ELISA exhibited a sensitivity of 94.03% and a specificity of 100%, showing a high overall agreement rate of 95.83% and satisfactory consistency with IFA (Kappa value of 0.905).

**Table 2:** Results of rCap-ELISA and IFA applied to clinical pig serum samples.

		IFA		
		Positive	Negative	Total
rCap-ELISA	Positive	63	0	63
	Negative	4	29	33
	Total	67	29	96
	Agreement	94.02%	100%	95.83%

### Detection clinical swine sera via rCap-ELISA

**Figure 5:**
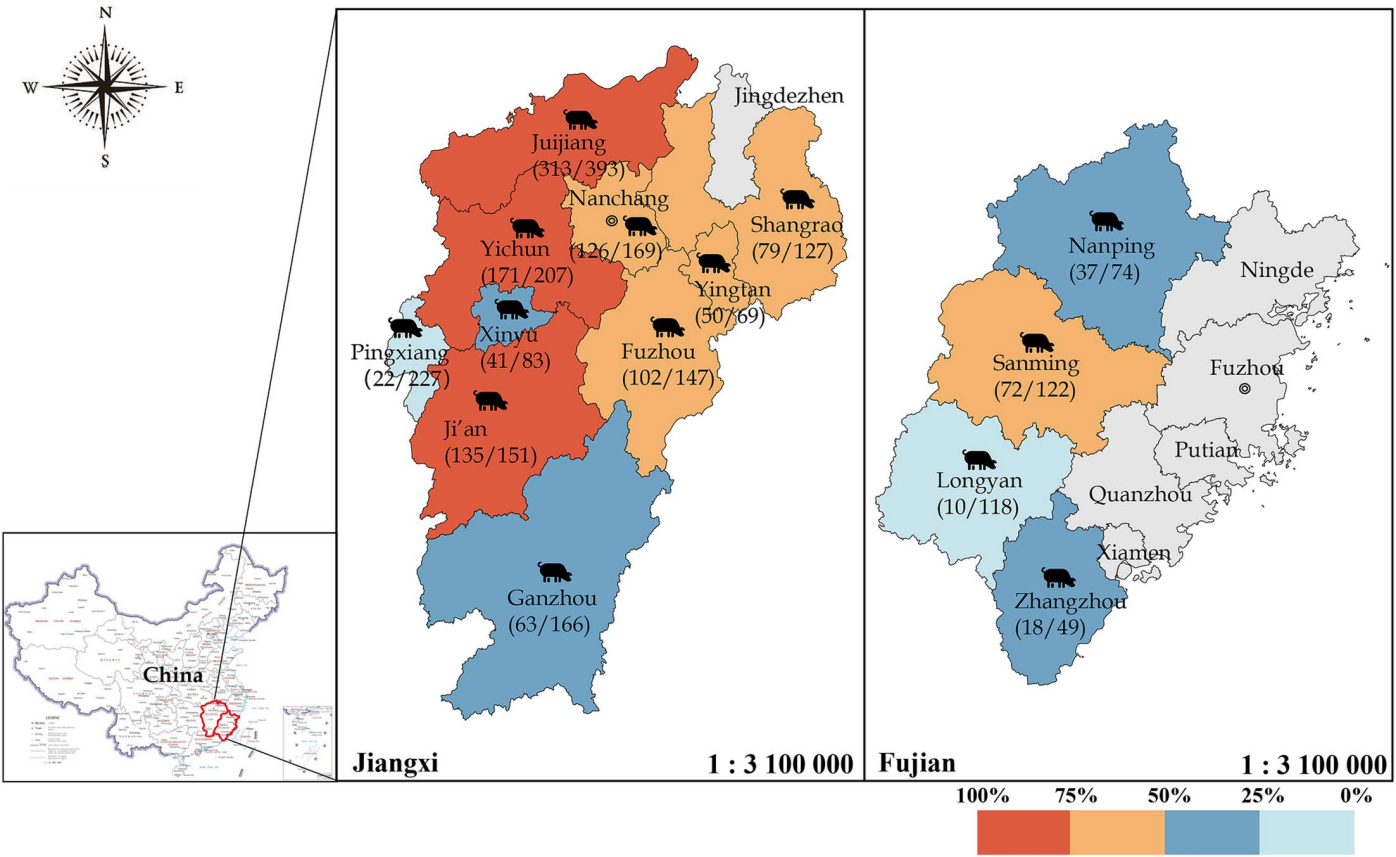
Seroprevalence of GETV in domestic swine in Jiangxi and Fujian provinces, China. Different colors represent varying levels of the positive rate, with gray indicating districts where no samples were collected. A total of 2,102 domestic swine samples from Fujian and Jiangxi Provinces were collected, and IgG antibodies against GETV were detected via rCap-ELISA.

The total positive rate of IgG antibodies against GETV was 37.74% in Fujian pigs and 63.36% in Jiangxi pigs (*p* < 0.01) according to an aeroepidemiologic investigation of domestic swine. The positive rates in the cities of Fuzhou, Ganzhou, Ji’an, Jiujiang, Nanchang, Shangrao, Xinyu, Yichun, Yingtan, and Pingxiang in Jiangxi province were 69.39, 37.95, 89.40, 79.64, 74.56, 62.20, 49.40, 82.61, 72.46, and 9.69%, respectively. In the cities of Longyan, Nanping, Sanming, and Zhangzhou in Fujian province, the rates were 8.47, 50.00, 59.02, and 36.73%, respectively (Figure 5). The number of positive pig farms in Jiangxi reached 32, accounting for 96.9% (32/33) of the total. Notably, 24 farms had a positive rate of ≥50%, with some reaching 100%.

## Discussion

The *Getah virus* (GETV) demonstrates strong adaptive capabilities, expanding its distribution through continuous mutation and evolution (Li et al., 2017). Consequently, the number of infected hosts, primarily horses and pigs, has increased. GETV has caused significant outbreaks in countries such as Japan, India, and China, leading to substantial economic losses in the equine and porcine industries. This virus poses a considerable challenge to global public health.

Currently, numerous studies on ELISA detection methods have been conducted, with most employing indirect ELISA using E2 proteins due to their stable conformation and minimal variation. One study utilized the complete E2 protein as an antigen to establish an ELISA method (Bannai et al., 2019; Bannai et al., 2020). Another study used GETV viral particles as antigens to develop an indirect ELISA method for detecting antibodies in pigs and horses (Lee et al., 2016). Although the direct use of purified viral particles offers good immunogenicity, it is complex to obtain, requires live cell culture, and may pose biosafety risks. An onsite immunochromatographic strip (ICS) using a recombinant E2 protein was successfully developed to detect GETV antibodies in horses (Zhong et al., 2024). Most current ELISA studies focus on the E2 domain, whereas investigations on the Cap domain are infrequent. This study employed recombinant Cap protein expressed in prokaryotes, known for its high immunogenicity, cost-effectiveness, and safety, enabling large-scale production in a short period. By optimizing the *Escherichia coli* cultivation temperature and time, the most favorable conditions were 16°C for 16 h. Under these conditions, the recombinant protein rCap was predominantly expressed in a non-inclusion body form, significantly increasing protein yield and activity, and simplifying experimental procedures. Animal experiments demonstrated that the refolded recombinant protein rCap has good immunogenicity and is highly suitable as an antigen for detecting anti-GETV antibodies.

The checkerboard titration method was used to optimize various reaction conditions, and 100 clinical pig serum samples were tested to plot the ROC curve. The area under the ROC curve (AUC) was 0.9578, indicating the good accuracy of the rCap-ELISA. Based on a sensitivity of 0.84 and a specificity of 0.92, the maximum Youden index was calculated to be 0.76, with a corresponding cutoff of 0.45, which is similar to other studies detecting pig GETV antibodies by ELISA (0.344 ~ 0.385) (Kuwata et al., 2018; Bannai et al., 2019; Sun et al., 2022). A higher cutoff may result in some weak positives being misjudged as negatives. However, our tests revealed that positive samples with an initial OD_450_ value of 1.5 remained positive even after a 1,600-fold dilution. Therefore, the high cutoff determined by this method does not significantly affect the accuracy of the results but helps avoid false positives due to nonspecific binding, thereby increasing the accuracy of the test results. Given the high sensitivity of the ELISA method, the high OD_450_ values (0.4–0.45) may be caused by trace amounts of anti-GETV IgG antibody molecules remaining in pigs. Other detection methods with good sensitivity, such as qPCR, LAMP, and IFA, can be used for further confirmation. Additionally, the intra-assay and inter-assay coefficients of variation for this method were 5.68–9.60% and 6.34–9.21%, respectively, confirming the good reproducibility of the method.

Some studies have reported the detection of GETV antibodies in pigs: 23.1% in Thailand (Rattanatumhi et al., 2022), 16.0% in wild boars in Japan (Kuwata et al., 2018), and 37.1% in Sichuan, China (You et al., 2024). These findings indicate that GETV has widely spread among pig populations in East Asian and Southeast Asian countries. The positive rates of GETV antibodies were significantly higher in pigs and horses than in other species, with positive rates of 93% in horses in Japan (Imagawa et al., 1981), 20.25% in bovines in the China-Myanmar border area (Liu et al., 2023), 10.0% in sheep, and 25.1% in cattle in China (Shi et al., 2022). To verify the reliability of the rCap-ELISA and understand the prevalence of GETV in southern China, we surveyed 2,102 samples from 42 pig farms in Jiangxi and Fujian provinces, China. The overall positive rates in Jiangxi and Fujian were 63.36 and 37.74%, respectively (*p* < 0.01), likely due to varying mosquito densities. GETV is an arthropod-borne virus primarily transmitted by mosquitoes, with higher mosquito densities reported in Jiangxi than in Fujian. Significant differences in positive rates were observed among different areas in Jiangxi, with Jiujiang, Yichun, and Ji’an having notably higher rates than Shangrao, Fuzhou, Pingxiang, Xinyu, and Ganzhou (p < 0.01). In Fujian, the positive rates in Sanming and Nanping were higher than those in Longyan and Zhangzhou (*p* < 0.01), indicating a clear decreasing trend from west to east. This suggests that GETV may have initially become prevalent among pigs in Jiujiang, Yichun, and Ji’an in Jiangxi before spreading to surrounding areas. This further confirms that GETV is rapidly spreading among pigs in southern China, warranting close attention and necessary preventive measures to prevent further spread.

Additionally, the detection and analysis of various pig types revealed that gilts and pregnant sows presented the highest rate of positive GETV antibody incidence, whereas piglets presented the lowest rate. This study suggests that sows’ immune systems have had time to form immunological memory since they are exposed to the virus for a longer period compared to piglets. When the virus resurfaces, this memory facilitates quicker antibody production. On the other hand, the majority of the pigs in our study had early exposure to the virus. Despite their normal clinical symptoms, their immune systems failed to produce the necessary antibodies in a timely manner.

The high positive rates in some farms might be influenced by factors such as selection bias from samples originating from diseased pig populations and the small sample size in Fujian, which may introduce significant randomness. However, the results of this study demonstrate that GETV has been widely disseminated in Jiangxi and Fujian, China, and has a tendency to spread to other regions.

## Conclusion

The recombinant protein rCap from GETV was used in this study to construct an indirect ELISA approach. This technique may aid in the development of a reliable method for the widespread identification of GETV in pigs. Additionally, this work provides a foundation for future research on the prevalence of GETV and highlights the need for more frequent monitoring of GETV in pigs from Jiangxi and Fujian, China.

## Data Availability

The original contributions presented in the study are included in the article/Supplementary material, further inquiries can be directed to the corresponding author.

## Supplementary material

SUPPLEMENTARY FIGURE S1 The circular map illustrates the GETV-Cap-His fusion expression plasmid. The Cap domain (804 bp) was fused at the 3′ terminus with a 6 × His sequence and subsequently inserted into the EcoR I-Xho I restriction sites of the prokaryotic expression vector pET-32a (+), resulting in the recombinant plasmid pET-32a-Cap.

SUPPLEMENTARY FIGURE S2 Optimization of rCap-ELISA working conditions. Optimization of concentration of blocking time **(A)**, chromogenic time of TMB **(B)**, incubating time of antisera **(C)**, and second antibody incubation time **(D)**.
