# Supplementary figures and images for: Seroprevalence of Getah virus in pigs in Southeast China determined with a recombinant Cap protein-based indirect ELISA
Source: Front Microbiol. 2025 Feb 17;16:1547670. doi: 10.3389/fmicb.2025.1547670 (PMC11872902; doi:10.3389/fmicb.2025.1547670)

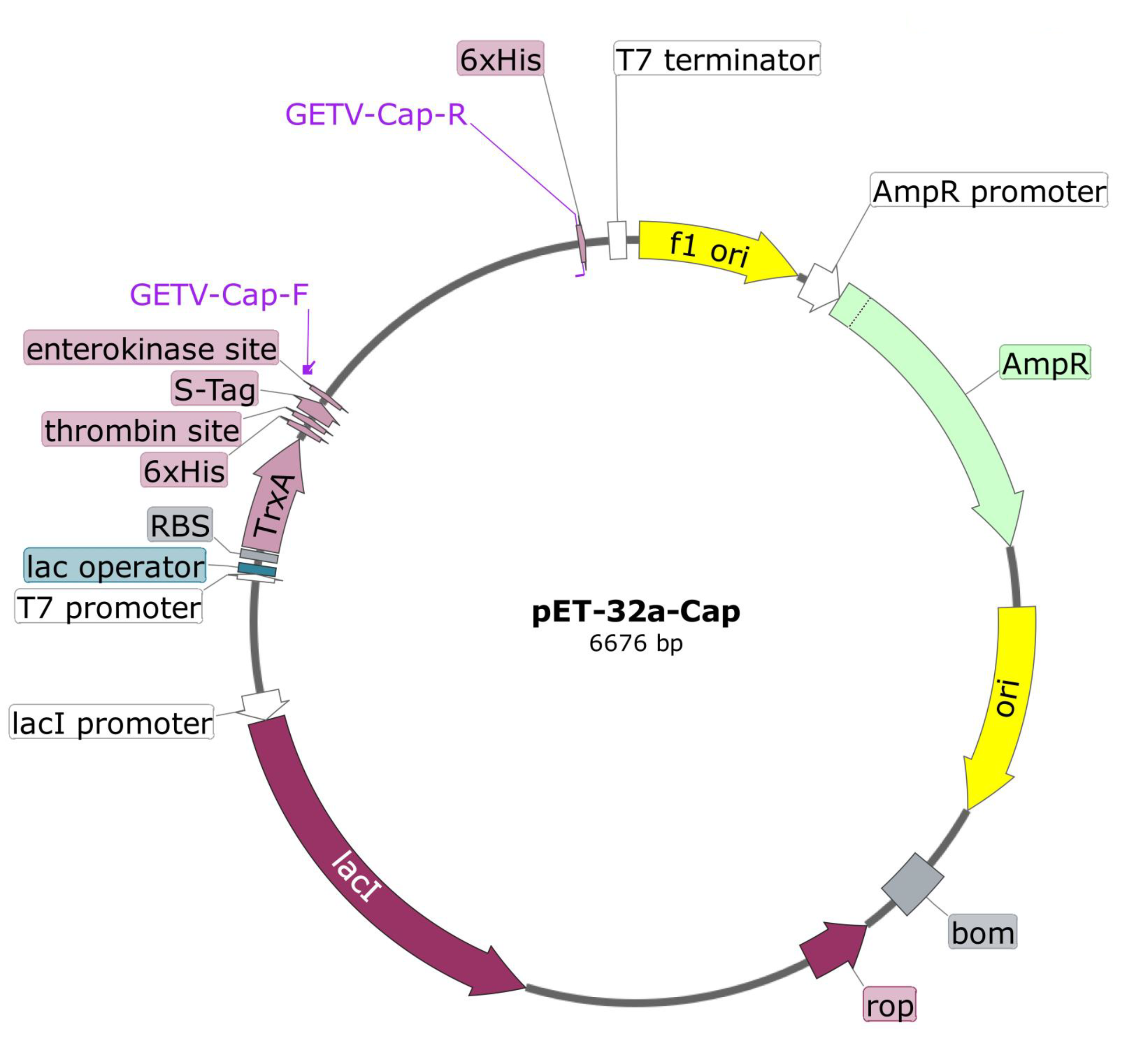

Supplement: SUPPLEMENTARY FIGURE S1 — The circular map illustrates the GETV-Cap-His fusion expression plasmid. The Cap domain (804 bp) was fused at the 3′ terminus with a 6 × His sequence and subsequently inserted into the EcoR I-Xho I restriction sites of the prokaryotic expression vector pET-32a (+), resulting in the recombinant plasmid pET-32a-Cap. [file Image_1.tif]

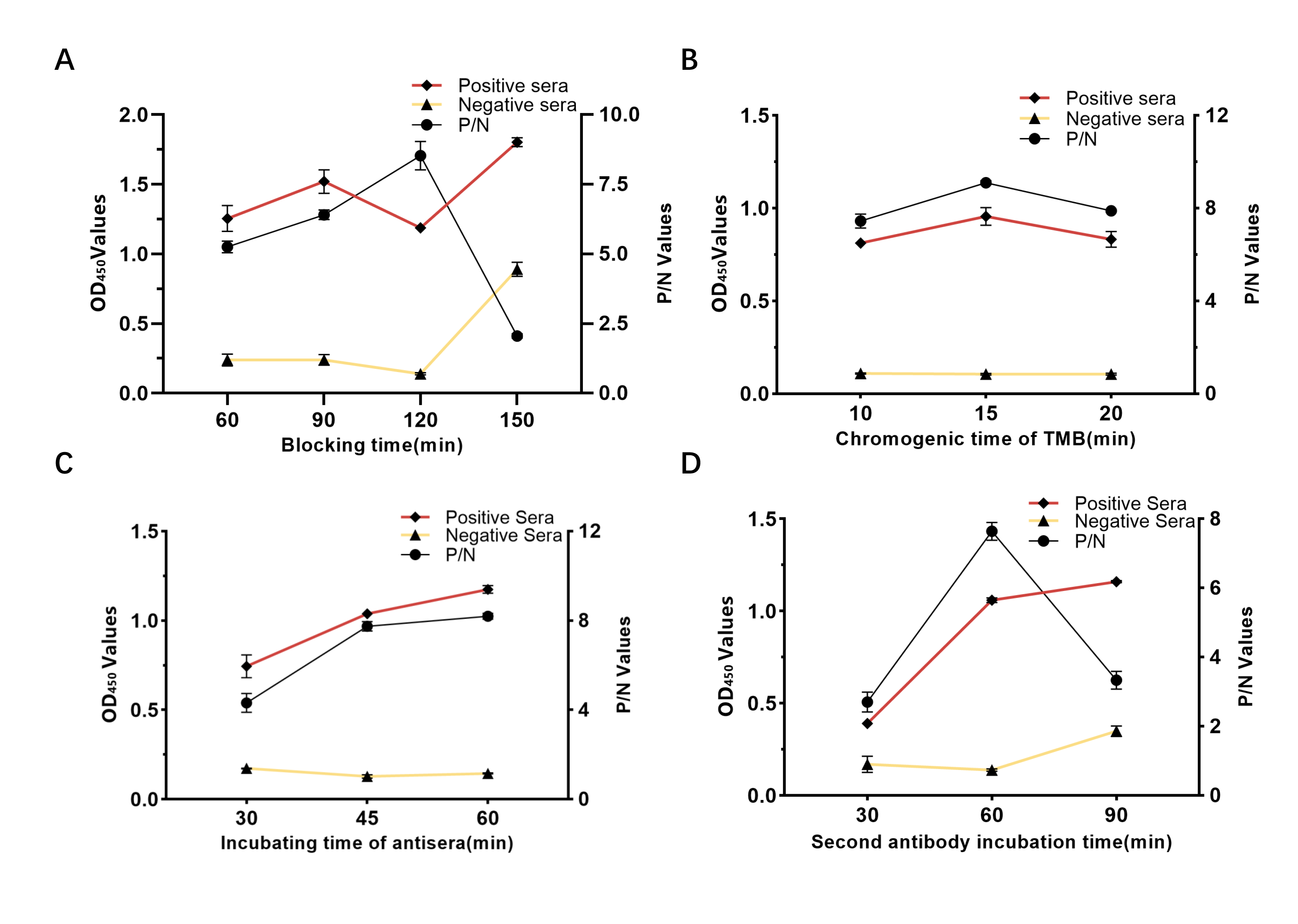

Supplement: SUPPLEMENTARY FIGURE S2 — Optimization of rCap-ELISA working conditions. Optimization of concentration of blocking time (A), chromogenic time of TMB (B), incubating time of antisera (C), and second antibody incubation time (D). [file Image_2.tif]
