# Supplementary material for: Seroprevalence of Getah virus in pigs in Southeast China determined with a recombinant Cap protein-based indirect ELISA
Source: Front Microbiol. 2025 Feb 17;16:1547670. doi: 10.3389/fmicb.2025.1547670 (PMC11872902; doi:10.3389/fmicb.2025.1547670)
Supplement: Supplementary file 3 [file Table_1.docx]

**Table S1.** Background information of samples collected in this study.

| Pig Farm | City, Province | Sample Number | Positive Sample | Positive Rate |
| --- | --- | --- | --- | --- |
| Farm No.1 | Jiujiang, Jiangxi | 41 | 40 | 97.56% |
| Farm No.2 | Nanchang, Jiangxi | 52 | 43 | 82.69% |
| Farm No.3 | Jiujiang, Jiangxi | 30 | 30 | 100% |
| Farm No.4 | Jiujiang, Jiangxi | 20 | 18 | 90.00% |
| Farm No.5 | Jiujiang, Jiangxi | 30 | 28 | 93.99% |
| Farm No.6 | Jiujiang, Jiangxi | 30 | 29 | 96.99% |
| Farm No.7 | Jiujiang, Jiangxi | 30 | 23 | 76.66% |
| Farm No.8 | Jiujiang, Jiangxi | 29 | 22 | 75.86% |
| Farm No.9 | Jiujiang, Jiangxi | 29 | 29 | 100% |
| Farm No.10 | Jiujiang, Jiangxi | 30 | 21 | 70.00% |
| Farm No.11 | Jiujiang, Jiangxi | 30 | 5 | 16.67% |
| Farm No.12 | Jiujiang, Jiangxi | 29 | 9 | 31.03% |
| Farm No.13 | Jiujiang, Jiangxi | 39 | 33 | 84.62% |
| Farm No.14 | Yingtang, Jiangxi | 69 | 50 | 72.46% |
| Farm No.15 | Nanchang, Jiangxi | 40 | 39 | 97.50% |
| Farm No.16 | Longyan, Fujian | 50 | 3 | 6.00% |
| Farm No.17 | Xinyu, Jiangxi | 83 | 41 | 49.40% |
| Farm No.18 | Ganzhou, Jiangxi | 38 | 0 | 0 |
| Farm No.19 | Jiujiang, Jiangxi | 26 | 26 | 100.00% |
| Farm No.20 | Ji'an, Jiangxi | 68 | 52 | 76.47% |
| Farm No.21 | Shangrao, Jiangxi | 64 | 49 | 76.56% |
| Farm No.22 | Ji'an, Jiangxi | 83 | 83 | 100.00% |
| Farm No.23 | Yichun, Jiangxi | 100 | 90 | 90.00% |
| Farm No.24 | Ganzhou, Jiangxi | 47 | 29 | 61.70% |
| Farm No.25 | Fuzhou, Jiangxi | 31 | 28 | 90.32% |
| Farm No.26 | Sanmin, Fujian | 73 | 24 | 32.87% |
| Farm No.27 | Nanchang, Jiangxi | 77 | 44 | 57.14% |
| Farm No.28 | Nanping, Fujian | 50 | 33 | 66.00% |
| Farm No.29 | Zhangzhou, Fujian | 30 | 8 | 26.67% |
| Farm No.30 | Sanmin, Fujian | 49 | 48 | 97.87% |
| Farm No.31 | Nanping, Fujian | 24 | 4 | 16.67% |
| Farm No.32 | Shangrao, Jiangxi | 9 | 7 | 77.78% |
| Farm No.33 | Yichun, Jiangxi | 10 | 5 | 50.00% |
| Farm No.34 | Yichun, Jiangxi | 97 | 76 | 78.35% |
| Farm No.35 | Ganzhou, Jiangxi | 81 | 34 | 41.98% |
| Farm No.36 | Fuzhou, Jiangxi | 43 | 27 | 62.79% |
| Farm No.37 | Longyan, Fujian | 50 | 6 | 12.00% |
| Farm No.38 | Shangrao, Jiangxi | 54 | 23 | 42.59% |
| Farm No.39 | Zhangzhou, Fujian | 19 | 10 | 52.63% |
| Farm No.40 | Fuzhou, Jiangxi | 73 | 47 | 64.38% |
| Farm No.41 | Longyan, Fujian | 18 | 1 | 5.56% |
| Farm No.42 | Pingxiang, Jiangxi | 227 | 22 | 9.69% |
